# The predictive value of cardiac MRI strain parameters in hypertrophic cardiomyopathy patients with preserved left ventricular ejection fraction and a low fibrosis burden: a retrospective cohort study

**DOI:** 10.3389/fcvm.2023.1246759

**Published:** 2023-09-13

**Authors:** Alireza Salmanipour, Amir Ghaffari Jolfayi, Nazanin Sabet Khadem, Nahid Rezaeian, Hamid Chalian, Saeideh Mazloomzadeh, Sara Adimi, Sanaz Asadian

**Affiliations:** ^1^ Rajaie Cardiovascular Medical and Research Center, Iran University of Medical Sciences, Tehran, Iran; ^2^ Department of Radiology, School of Medicine, Iran University of Medical Sciences, Tehran, Iran; ^3^ Department of Radiology, Cardiothoracic Imaging, University of Washington, Seattle, WA, United States

**Keywords:** hypertrophic cardiomyopathy (HCM), cardiac MRI, feature tracking, cardiac function, adverse events

## Abstract

**Background:**

Prompt interventions prevent adverse events (AE) in hypertrophic cardiomyopathy (HCM). We evaluated the pattern and the predictive role of feature tracking (FT)-cardiac magnetic resonance (CMR) imaging parameters in an HCM population with a normal left ventricular ejection fraction (LVEF) and a low fibrosis burden.

**Methods:**

The CMR and clinical data of 170 patients, consisting of 142 HCM (45 ± 15.7 years, 62.7% male) and 28 healthy (42.2 ± 11.26 years, 50% male) subjects, who were enrolled from 2015 to 2020, were evaluated. HCM patients had a normal LVEF with a late gadolinium enhancement (LGE) percentage below 15%. Between-group differences were described, and the potent predictors of AE were determined. A *P*-value below 0.05 was considered significant.

**Results:**

LV global longitudinal, circumferential, and radial strains (GLS, GCS, and GRS, respectively) and the LV myocardial mass index (MMI) were different between the healthy and HCM cases (all *P*s < 0.05). Strains were significantly impaired in the HCM patients with a normal MMI. A progressive decrease in LVGLS and a distinct fall in LVGCS were noted with a rise in MMI. AE were predicted by LVGLS, LVGCS, and the LGE percentage, and LVGCS was the single robust predictor (HR, 1.144; 95% CI, 1.080–1.212; *P *= 0.001). An LVGCS below 16.2% predicted AE with 77% specificity and 58% sensitivity.

**Conclusions:**

LV strains were impaired in HCM patients with a normal EF and a low fibrosis burden, even in the presence of a normal MMI. CMR parameters, especially FT-CMR values, predicted AE in our HCM patients.

## Introduction

Hypertrophic cardiomyopathy (HCM) is an inherited disorder characterized by left ventricular (LV) hypertrophy and is unexplainable by other causes (1–3). HCM is the most common monogenic cardiovascular disorder, with an estimated prevalence of 1:250–500 in the adult population, predominantly affecting adolescents and young adults while rare in children (4–6). The LV ejection fraction (EF), as an index of systolic function, often remains within the normal range in HCM despite disease progression (7).

Myocardial fibers are arranged in 3 different orientations as a continuum of 2 helical geometries, helping amplify myocyte contraction and cardiac function as a single unit. This superstructure deteriorates in HCM, resulting in faulty mechanics despite an apparently preserved EF. HCM diagnosis and characterization are based on echocardiography and magnetic resonance imaging (MRI), although cardiac magnetic resonance imaging (CMR) provides better identification and risk stratification by detecting *in vivo* fibrosis (8).

Several studies have examined imaging features that could better elucidate myocardial abnormalities and patient outcomes (9–12). One of these methods is the myocardial strain analysis by CMR to assess subclinical function impairment. Strains are reported in 3 directions: longitudinal, circumferential, and radial. The longitudinal strain represents subendocardial fiber deformation, while circumferential and radial strains reveal mid-myocardial and subepicardial fiber changes, respectively (13). Multiple parameters can affect LV myocardial strains in patients with HCM, including LVEF, the myocardial mass, the myocardial fibrosis burden, and the left ventricular outflow tract (LVOT) obstruction (14).

In the present study, utilizing the feasible cardiac magnetic resonance feature-tracking (CMR-FT) method, we aimed to define the myocardial strain pattern in HCM patients. Moreover, we investigated the role of CMR parameters in the prognostication of HCM patients with a normal LVEF and a low fibrosis burden.

## Methods

The institutional research committee approved this study and waived the need for informed consent due to the retrospective design of this study.

### Study population

The current investigation retrospectively enrolled 241 patients with HCM who underwent CMR between 2015 and 2020 in our institution. Additionally, the CMR findings of 28 healthy volunteers were retrieved from the center's normal CMR examination archives. Healthy subjects had a normal physical examination, no personal or family history of cardiac disease, and no cardiovascular risk factors, composed of hypertension, diabetes mellitus, and dyslipidemia.

### Diagnostic criteria

All patients with a definite diagnosis of HCM, according to the American heart association/American college of cardiology guidelines for diagnosing and treating patients with hypertrophic cardiomyopathy, were enrolled (15).

**Figure 1:**
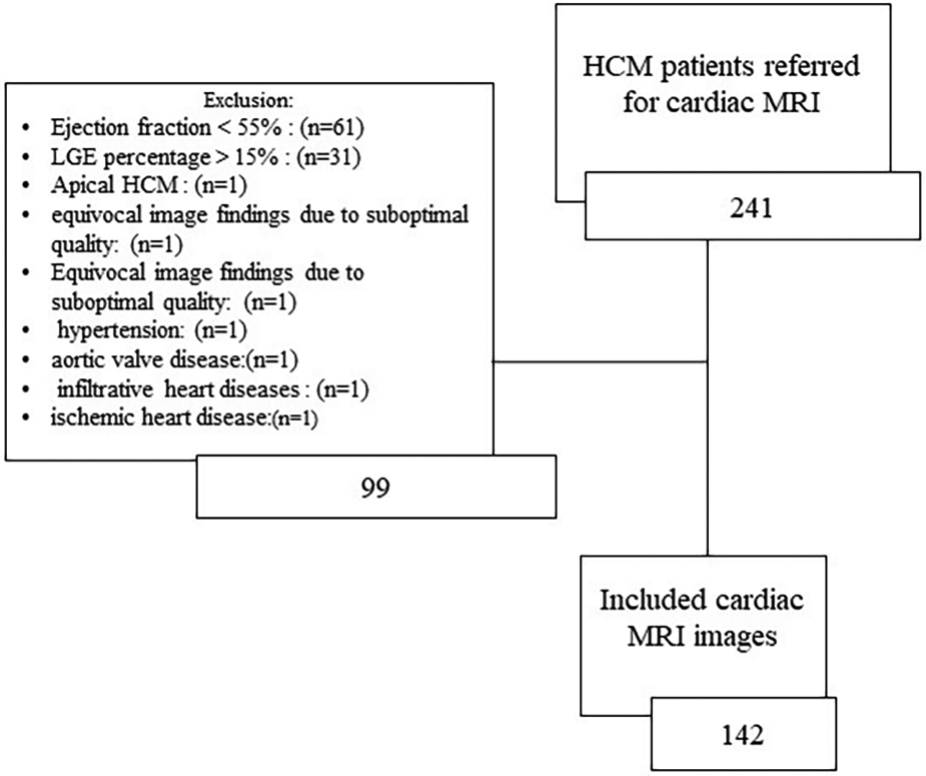
Diagram of the study population. HCM, hypertrophic cardiomyopathy; LGE, late gadolinium enhancement.

The exclusion criteria were an LVEF below 55%, a late gadolinium enhancement (LGE) percentage of more than 15%, hypertension, aortic valve disease, infiltrative heart diseases (e.g., Fabry disease, Danon disease, and cardiac amyloidosis), athlete's heart, ischemic heart disease, significant cardiac arrhythmias during CMR acquisition, and renal impairment (defined as an estimated glomerular filtration rate <30 ml/min precluding gadolinium injection). Also, other types of HCM, such as apical HCM, are excluded from the study. Furthermore, CMR studies that yielded equivocal findings due to suboptimal quality were excluded from the study (Figure 1).

### Study population classification

The medical records of the patients were reviewed. Then, based on their transthoracic echocardiography (TTE)-measured LVOT gradient, the patients were classified into 2 groups: an LVOT gradient of less than 50 mm Hg and an LVOT gradient of 50 mm Hg or higher. TTE examinations with a maximum interval of 6 months from the CMR examination were selected for analysis. The former group was regarded as a no or mild LVOT obstruction group and the latter as a severe LVOT obstruction group (16, 17).

In another classification, the patients were divided based on their myocardial mass index (MMI) into normal and increased MMI categories. An MMI exceeding 81 g/m^2^ for females and 85 g/m2 for males was regarded as increased (18).

### CMR

CMR images were acquired using a 1.5-T MRI equipment (Siemens Avanto, Erlangen, Germany) with an 8-element phased-array receiver surface coil. A semi-automatic post-processing program (CVi42; Circle Cardiovascular Imaging Inc, Calgary, Canada) was applied for the measurements.

### CMR function

Electrocardiography-gated cine steady-state free precession in the 2-, 3-, and 4-chamber views, the right ventricular and LV outflow tracts planes, and a stack of short-axis slices covering LV during breath-hold at end-expiration (slice thickness = 8 mm, the field of view = 300 mm, flip angle = 65°, bandwidth = 925 Hz/Px, imaging matrix = 156 × 192, and repetition time/echo time = 2.7/1.2 ms) were acquired. Parallel imaging was utilized. The endocardial and epicardial borders were manually drawn in short-axis end-diastolic and end-systolic images and propagated throughout all ventricular slices. Functional parameters, composed of the EF, end-diastolic and end-systolic volumes, the LV mass index, the maximal septal thickness, the presence of the systolic anterior motion of the mitral valve, and the ratio of the asymmetric septal hypertrophy, were registered.

### CMR-FT

**Figure 2:**
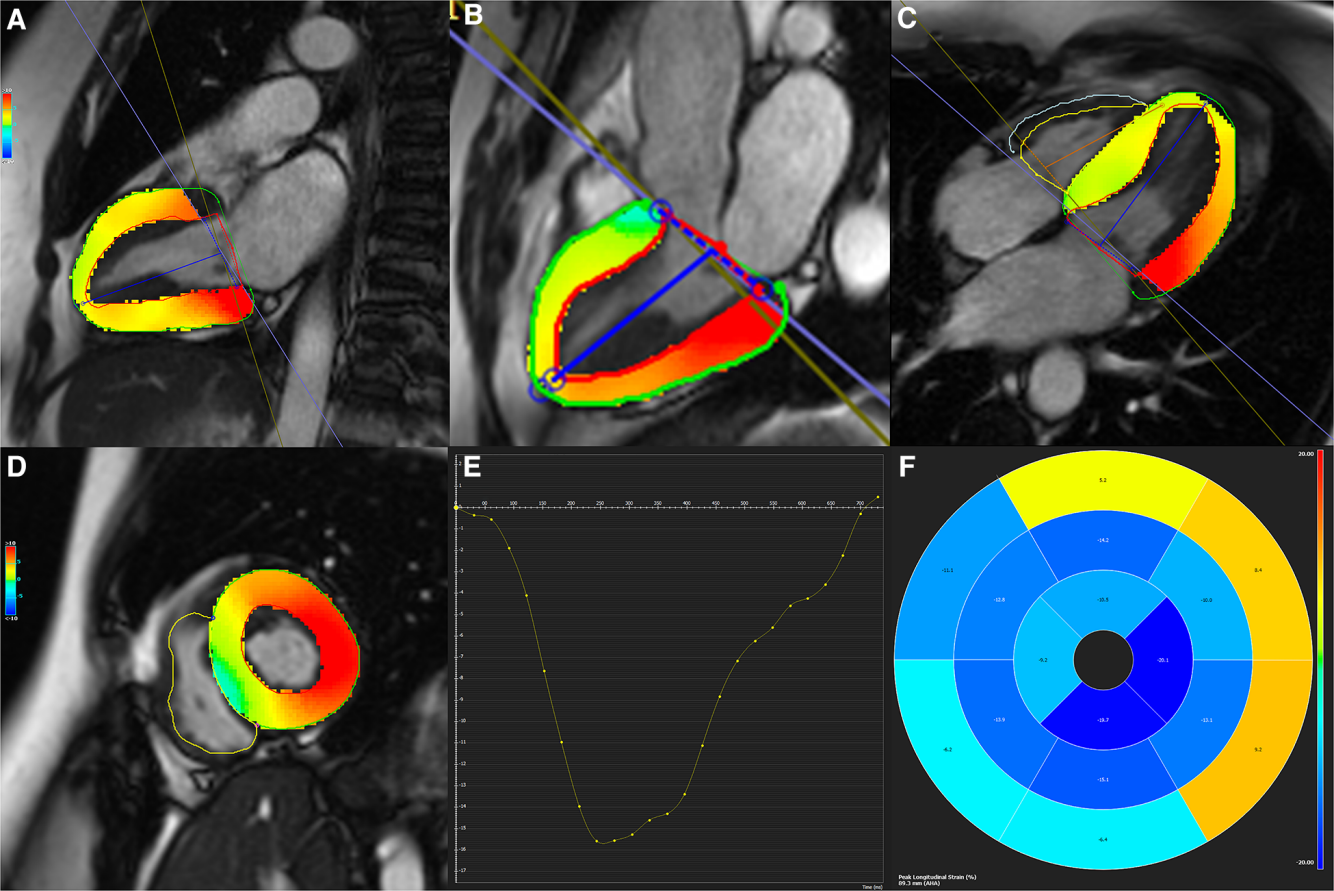
(**A**–**D**) Two-, three-, and four-chamber as well as short-axis cine functional sequences with defined endocardial and epicardial contours. (**E**) Strain curve, and (**F**) Bull's eye map are depicted for global circumferential and longitudinal strains.

LV end-diastolic and end-systolic frames of 2-, 3- and 4-chamber views and the short-axis plane were selected. Optimal brightness adjustment was done to ensure the best contrast to make accurate discrimination between the endocardium and the blood pool. The endocardial and epicardial contours were defined manually and propagated throughout the slices, and 3D LV strains, consisting of global longitudinal (GLS), global circumferential (GCS), and global radial (GRS) strains, were calculated utilizing the CMR-FT method (Figure 2). The absolute values of strains were utilized for analysis.

### LGE imaging

LGE images were obtained 15 min after the injection of the gadolinium contrast agent, gadotetrate meglumine (Dotarem), applying the phase-sensitive inversion recovery sequence. Breath-held segmented single-shot protocol (slice thickness = 8 mm, the field of view = 320 mm, flip angle = 40°, bandwidth = 1,445 Hz/Px, imaging matrix = 192 × 192, and repetition time/echo time = 2.9/1.1 ms) with selecting the inversion time to null the normal myocardium (typically 200–250 ms) was applied. Considering a 5-standard deviation from the mean myocardial signal intensity, the LGE percentage was measured. The results were assessed visually and modified if needed.

### LVOT evaluation

Based on echocardiographic findings obtained from patients' medical records, the patients' LVOT gradients were collected. Gradient measurement was performed based on the modified Bernoulli equation.

On CMR, cine LVOT images in at least 5 consecutive slices were acquired. The presence of the systolic anterior motion of the mitral valve and LVOT turbulence were registered.

### Follow-up data

For each patient, we considered at least one follow-up. If a patient had several follow-ups, we considered the last one. A composite of adverse events, consisting of sudden cardiac death, aborted sudden cardiac death (unsuccessful cardiopulmonary resuscitation), implantable cardioverter defibrillator insertion, and deteriorated systolic function (an EF decline to <40%), was considered and registered.

### Data collection

CMR measures were registered by two experts with more than five years of expertise (a cardiologist and a radiologist) in cardiovascular imaging. Readers were blind to the study population's data. Interobserver variability was reported, and both examiners' consensus resolved any conflicts.

Echocardiographic, clinical, and follow-up data, including physician visits, lab data, and imaging examinations, were collected by reviewing patients' medical records and/or telephone calls whenever needed.

### Statistical analysis

SPSS version 22 (IBM incorporation) was utilized for statistical analysis. Categorical and continuous variables were reported as frequencies (percentages) and mean ± standard deviation (SD), respectively. The Kolmogorov–Smirnov test was utilized to assess the normality of distribution. Between-group comparisons were performed using the *t*, Mann–Whitney *U*, analysis of variances (ANOVA), and *χ*^2^ tests, whichever was appropriate. The *post hoc* test of the least significant difference described the pattern of the intergroup changes. Univariate and multivariate Cox regression analyses were applied to evaluate the role of CMR parameters in revealing undesirable outcomes. For the definition of the cutoff point, specificity, and sensitivity of the predictor variables, the receiver operating characteristic (ROC) curve was utilized. Moreover, *P*-values below 0.1 for the univariate Cox regression analysis and 0.05 for the rest of the tests were considered statistically significant.

## Results

### Study population characteristics

CMR examinations of 170 patients, consisting of 142 subjects with HCM (mean ± SD age =45 y ± 15.7; 62.7% male) and 28 healthy subjects (mean ± SD age =42.2y ± 11.26; 50% male), were included. Interobserver variability was estimated to be 6.3% and the examiners’ consensus-resolved conflicts.

The mean ± SD of the body surface area was 1.87 m^2^ ± 0.14, and the mean ± SD of MMI was 59.57 g/m^2^ ± 8.56 for the healthy subjects. No significant differences were observed in LVEF and end-diastolic and end-systolic volumes between the healthy population and the patients with HCM, whereas significant differences were noted between the 2 groups in LV MMI, LVGLS, LVGCS, and LVGRS (all *P*s < 0.05).

### HCM patients characteristics in LVOT groups

Table 1 presents the demographic characteristics and the baseline CMR data of the patients with HCM in the two LVOT groups.

**Table 1:** Demographics, baseline CMR characteristics, and follow-up data of the study population.

Variables	LVOT Gradient < 50mmHg (*n* = 69)		LVOT Gradient > 50mmHg (*n* = 73)		*P*-value
Demographics					
Age (y) (mean ± SD)	45.4 ± 16.55		44.6 ± 14.94		0.7
Gender *n* (%)	Male	Female	Male	Female	0.8
	44 (63.8%)	25 (36.2%)	45 (61.6%)	28 (38.4%)	
BSA (m^2^)	1.88 ± 0.24		1.85 ± 0.21		0.5
Positive family history	Negative	Positive	Negative	Positive	**0.03**
	38 (55%)	31 (45%)	24 (32.9%)	49 (67.1%)	
Diabetes	62 (90%)	7 (10%)	64 (87.7%)	9 (12.3%)	0.7
CMR findings					
LVEF (%) (mean ± SD)	61.4 ± 4		62.61 ± 4.6		0.1
LVEDVI (cc/m^2^) (mean ± SD)	70.72 ± 15.59		72.63 ± 14.72		0.4
LVESVI (cc/m^2^) (mean ± SD)	26.91 ± 6.52		27.38 ± 8.60		0.7
Main PA (mm) (mean ± SD)	23.96 ± 6.44		25.05 ± 4.50		0.2
Myocardial mass index (g/m^2^) (mean ± SD)	67.90 ± 20.24		79.75 ± 27.45		**0.003**
Maximum septal diameter (mm) (mean ± SD)	17.48 ± 4.19		20.49 ± 4.34		**0.001**
ASH ratio	2.80 ± 1.10		3.05 ± 1.23		0.2
LGE percentage (mean ± SD)	6.43 ± 4.17		6.37 ± 3.76		0.9
LVGLS (mean ± SD)	14.21 ± 2.80		14.41 ± 2.86		0.6
LVGCS (mean ± SD)	17.30 ± 2.78		17.88 ± 3.25		0.2
LVGRS (mean ± SD)	47.34 ± 14.03		48.54 ± 15.88		0.6
Increased Myocardial Mass index	Negative	Positive	Negative	Positive	0.06
	56 (81.2%)	13 (18.8%)	48 (65.8%)	25 (34.2%)	
SAM	55 (79.7%)	14 (20.3%)	2 (2.7%)	71 (97.3%)	**0.001**
Follow up data					
Poor outcome	59 (85.5%)	10 (14.4 %)	60 (82.2%)	13 (17.8%)	0.2
Sudden cardiac death	68 (98.6%)	1 (1.4%)	72 (98.7%)	1 (1.3%)	0.9
Aborted sudden cardiac death	69 (100%)	0 (0%)	72 (98.7%)	1 (1.3%)	0.5
ICD insertion	63 (91.3%)	6 (8.7 %)	64 (87.7%)	9 (12.3%)	0.3
Follow up Systolic dysfunction	65 (94.2%)	4 (5.8%)	71 (97.2%)	2 (2.8%)	0.6

CMR: cardiac magnetic resonance, LVOT: left ventricle outflow tract, BSA: body surface area, LV: left ventricle, EF: ejection fraction, EDVI: end-diastolic volume index, ESVI: end-systolic volume index, PA: pulmonary artery, ASH: asymmetric septal hypertrophy, LGE: late gadolinium enhancement, GLS: global longitudinal strain, GCS: global circumferential strain, GRS: global radial strain, SAM: systolic anterior motion, ICD: implantable cardioverter defibrillator

The difference in mean strain values was significant between the healthy controls and the HCM group. However, the mean strain values were not different between the 2 LVOT gradient groups (*P *> 0.05).

### CMR-FT parameters in MMI groups

**Figure 3:**
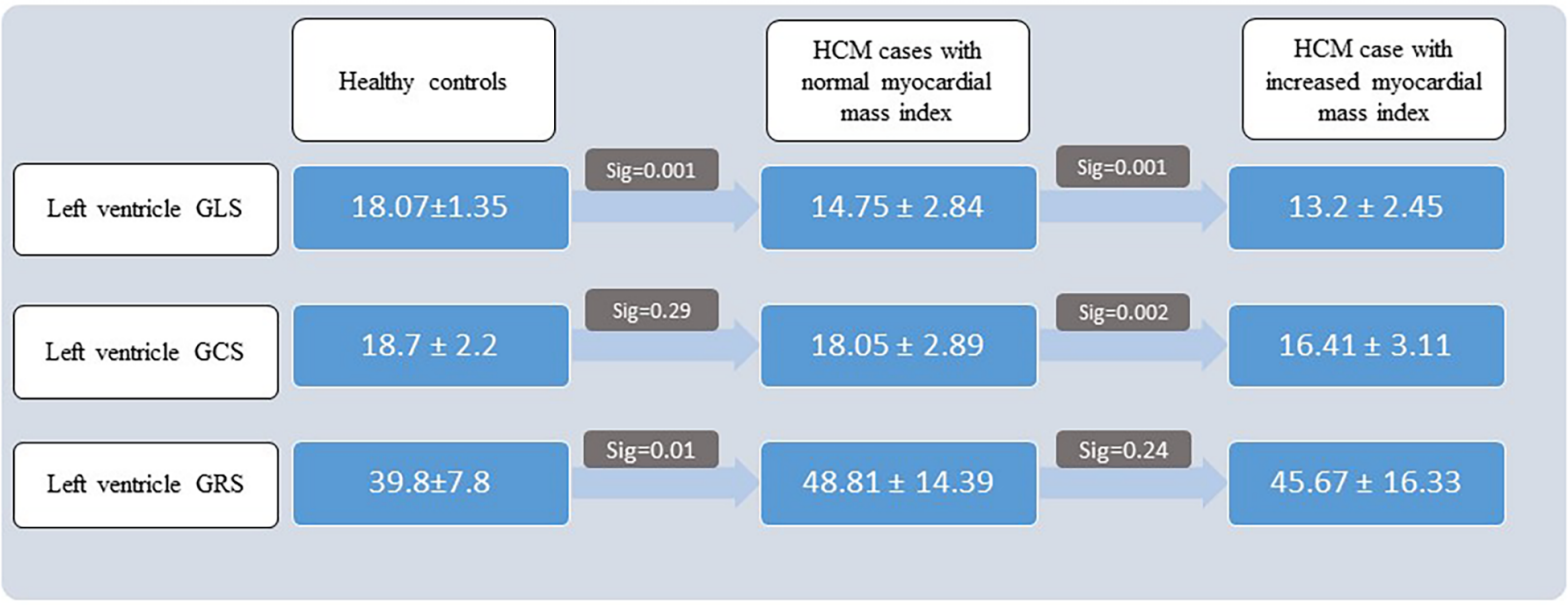
Results of *post hoc* least significant difference test. HCM, hypertrophic cardiomyopathy; GLS, global longitudinal strain; GCS, global circumferential strain; GRS, global radial strain.

The 1-way ANOVA revealed a significant difference in all 3 strain parameters between the healthy controls and the HCM cases with normal and increased myocardial mass (all *P*s < 0.05). Between-group changes were evaluated by applying the *post hoc* least significant difference test, and the results are depicted in Figure 3.

### The correlation between strain values and MMI

**Figure 4:**
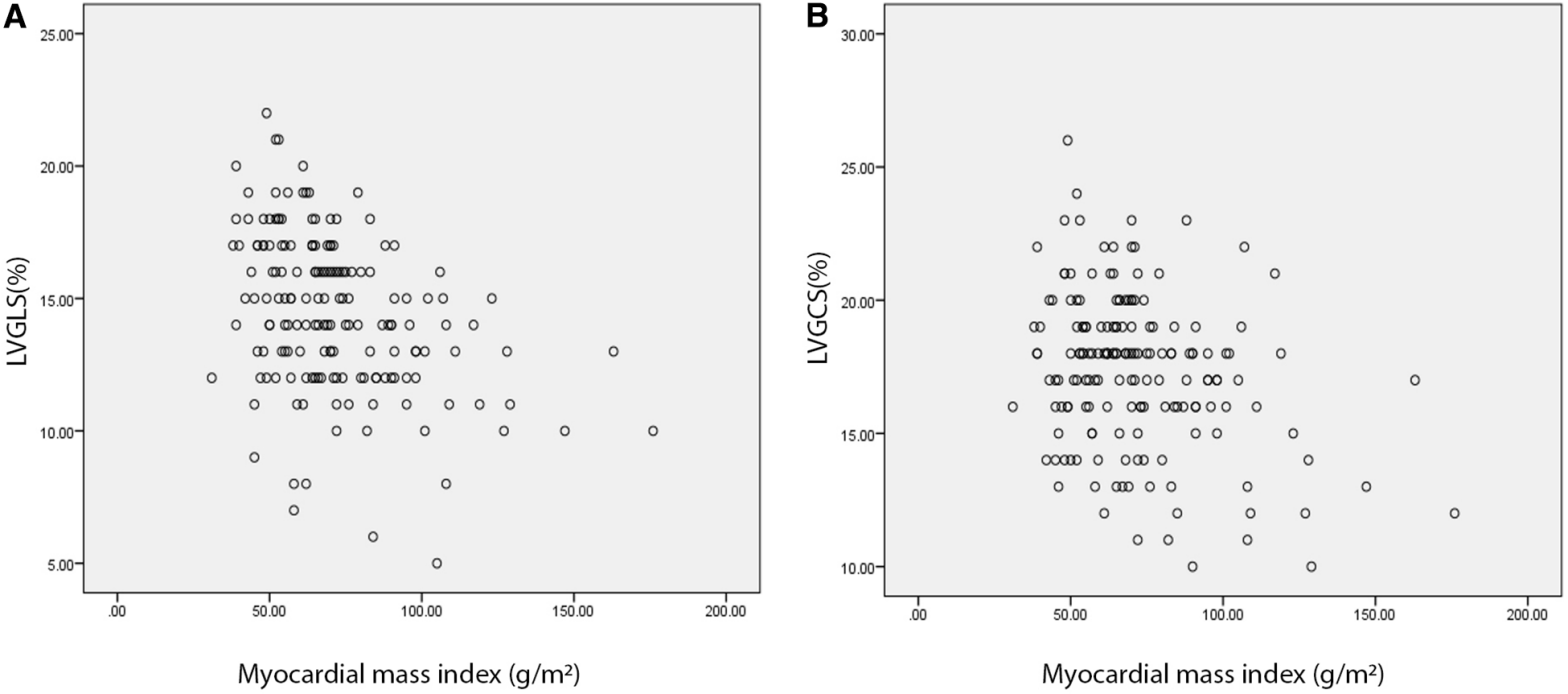
Scatter plots demonstrate a weak inverse correlation of absolute LVGLS (**A**) and LVGCS (**B**) values with myocardial mass index. Strain values are revealed on vertical and myocardial mass index on horizontal axes. LVGLS, left ventricle global longitudinal strain; LVGCS, left ventricle global circumferential strain.

The Pearson correlation test revealed a moderate inverse linear correlation between MMI and LVGLS and LVGCS (*r *= −0.4 and *r *= −0.32, respectively; *P*s = 0.001). Scatter plots are demonstrated in Figure 4.

### Follow-up data

The median (interquartile range) follow-up time was 25 months (23). Twenty-three patients developed adverse events. The results of the independent t-test analysis are depicted in Table 2. It was revealed that absolute values of LVGLS, LVGRS and LVGCS are considerably lesser in patients with adverse events than those without. Also, the myocardial mass index is significantly higher in patients with adverse events compared to others.

**Table 2:** Global strains and myocardial mass index comparison among the two groups with and without adverse events.

Variables	Patients with adverse events	Patients without adverse events	*P*-value
LVGLS	−12.649 ± 2.50	−14.473 ± 2.82	**0** **.** **005**
LVGRS	41.943 ± 12.81	49.145 ± 15.11	**0**.**035**
LVGCS	−15.451 ± 3.52	−17.892 ± 2.79	**0**.**004**
Myocardial mass index	89.087 ± 31.48	71.025 ± 22.91	**0**.**002**

GLS, global longitudinal strain; GCS, global circumferential strain; GRS, global radial strain.

The statistically significant values are in bold.

The results of the Cox regression analyses are depicted in Table 3. Variables with *P*-values below 0.1, consisting of LVGLS, LVGCS, and the LGE percentage, were entered into the multivariate Cox regression. The results revealed that LVGCS was the single predictor of adverse events (HR, 1.144; 95% CI, 1.080–1.212; *P *= 0.001).

**Table 3:** Results of Cox regression analyses.

Variable	Univariate			Multivariate		
	HR	95% CI	*P*-value	HR	95% CI	*P*-value
LVGLS	1.164	1.051–1.290	**0** **.** **004**	** **	** **	** **
LVGCS	1.146	1.082–1.213	**0**.**001**	**1.144**	**1.080–1.212**	**0.001**
LVGRS	1.004	0.982–1.027	0.7			
LVOT gradient	1.034	0.456–2.347	0.9			
LV myocardial mass index	1.002	0.993–1.012	0.6			
LGE percentage	1.096	0.995–1.209	**0**.**06**	** **	** **	** **

LV, left ventricle; GLS, global longitudinal strain; GCS, global circumferential strain; GRS, global radial strain; LVOT, left ventricle outflow tract; LGE, late gadolinium enhancement; HR, hazard ratio; CI, confidence interval.

The statistically significant values are in bold.

**Figure 5:**
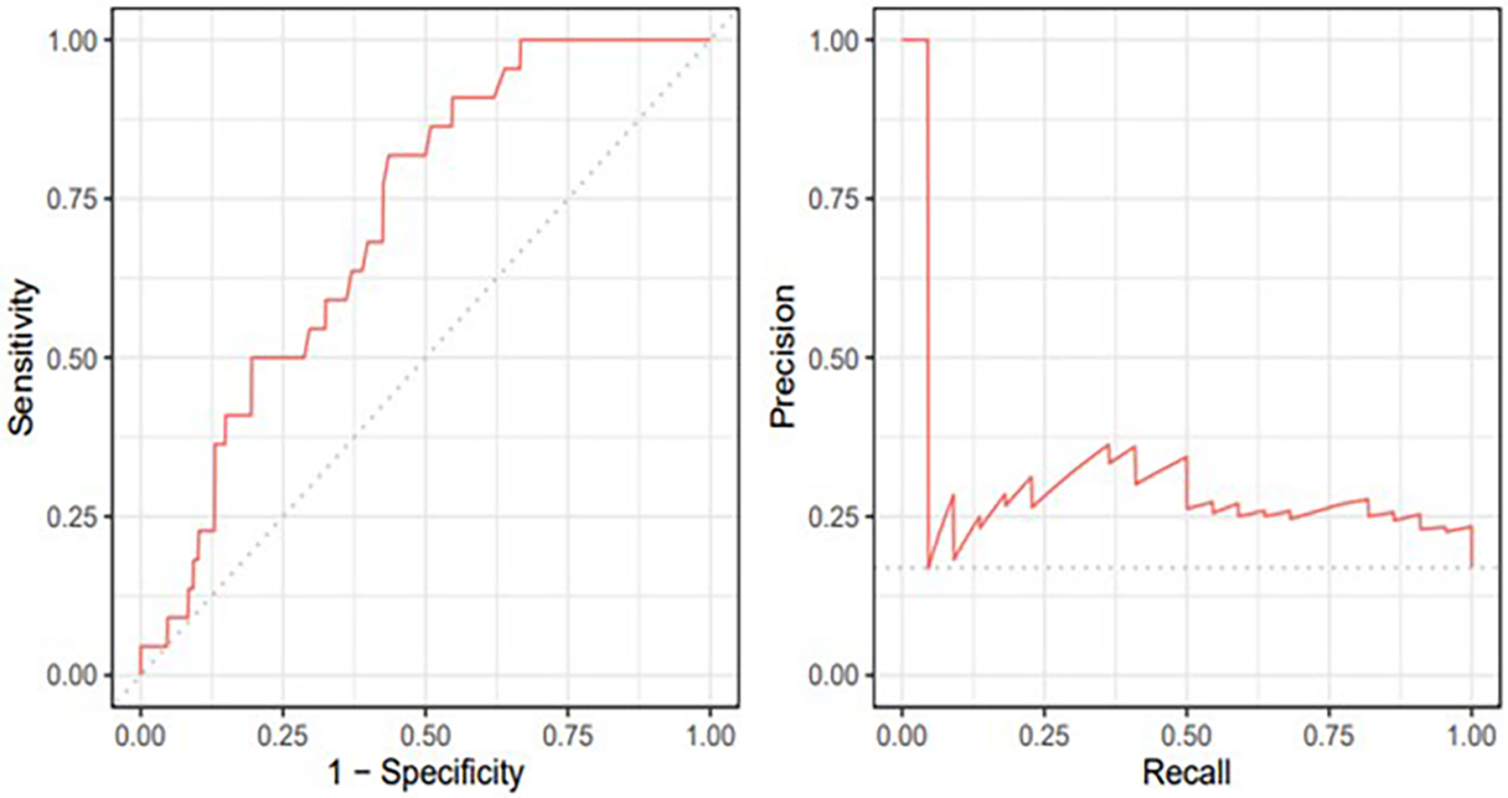
Receiver operating curve and precision-recall plots for left ventricle global circumferential strain.

The ROC analysis determined a cutoff point of 16.2% for LVGCS to predict adverse events with 77% sensitivity and 58% specificity (area under the curve = 0.716; *P *= 0.001) (Figure 5).

## Discussion

HCM is one of the prevalent causes of sudden cardiac death in young adults, hence the vital significance of its timely diagnosis and appropriate management strategy. In the present study, we investigated the CMR findings of 142 HCM patients with normal LV systolic function and a low myocardial fibrosis burden. Furthermore, we meticulously registered the follow-up data of the study population to determine whether CMR data could predict adverse events. Our principal findings are as follows:

A. The HCM and control groups were not statistically meaningfully different concerning LVEF and end-diastolic and end-systolic volumes, whereas the difference between them in all 3 global strain values and MMI constituted statistical significance.
B. In the HCM group, the severity of LVOT obstruction did not influence strain measurements.
C. LVGLS and LVGRS were impaired in the HCM group even if there was no increase in the myocardial mass. Along with an increase in the myocardial mass, a further decline in the LVGLS and LVGCS was detected.
D. Two of the strain values, LVGLS and LVGCS, and the LV myocardial LGE percentage predicted adverse events, with LVGCS being the single robust predictor.
E. An LVGCS value of 16.2% or less predicted adverse events with 77% sensitivity and 58% specificity.

Our results supported the notion that despite an unimpaired systolic function, abnormal myocardial deformation is present even without significant myocardial fibrosis. A previous study proved that CMR-FT parameters were impaired even in the carriers of HCM without the overt disease (19). Therefore, we suppose that CMR-derived strain values, influenced by myocardial fibers disarray, may reveal subclinical function impairment and assist in prompt cardioprotective treatment.

Multiple factors may affect the measurement of strain values (20–22). According to previous investigations, we assumed that an increased LVOT gradient, indicative of LVOT obstruction, might influence strain parameters (22). Nonetheless, our analysis did not prove this assumption, which may partly be due to our selected HCM population with an unimpaired LVEF and a low fibrosis burden. We believe that further investigations are needed in this regard.

Our study is one of the first investigations to address the mechanical changes secondary to an abnormal MMI. Our selection of HCM patients without systolic dysfunction and with a low fibrosis burden enabled us to evaluate the net effect of the LV myocardial mass to a great extent. We found a decreased LVGLS and an increased LVGRS in our selected HCM patients with normal myocardial mass. On the other hand, when the myocardial mass increases, LVGCS decreases distinctly. We suppose that it could be due to increased myocyte disarrangement in enlarged myocardial mass, which especially results in LVGCS decline. These findings are in agreement with previous studies suggesting that myocardial disarray in patients with HCM is a limiting factor of myocardial compliance and contractile function (23). Nevertheless, the precise myocardial mechanics in different myocardial mass groups have yet to be elucidated.

Remarkably, our results revealed a moderate inverse linear correlation between the net values of LVGLS and LVGCS and MMI. Strain values indicate function, especially in our selected population, who exhibited no decline in EF values. In a previous study, Dohy et al. showed that while there is no linear correlation between LVEF and MMI, LVGLS, as a functional parameter, correlated with MMI (24). Therefore, we suppose that strain values could be sensitive and early indicators of function impairment in HCM patients.

Previous studies have investigated the role of CMR parameters in indicating the outcome of patients with HCM (25–27). Parameters such as the LGE percentage, regional CMR-FT measures, and 3D global strains were reported to be capable of revealing outcomes in HCM subjects. Similarly, we demonstrated that the LGE percentage, LVGLS, and LVGCS were predictors of adverse events in our selected HCM patients. We also found that LVGCS was an independent predictor of adverse events and managed to define a cutoff point of 16.2% with 77% sensitivity and 58% specificity for LVGCS to reveal adverse events. Our findings are in line with previous investigations concluding that an LGE percentage exceeding 15% and an LVGLS decline are valuable in the risk stratification of patients suffering from HCM (28, 29). The discriminative characteristic of our study is that we selected HCM patients with normal systolic function and a low fibrosis burden and demonstrated that even in these benign-appearing clinical phenotypes, we might take advantage of CMR-FT parameters to estimate the probability of future adverse events.Morever, we utilized the CMR-FT method to determine strain values. This method is now established to have excellent reproducibility and agreement with the known MRI tagging and fast strain-encoded CMR imaging techniques. In contrast to other methods, CMR-FT does not require additional sequences and is, thus, more feasible in clinical practice (30).

In the present study, we observed that patients with a positive family history were more likely to show an increased LVOT gradient. It is noteworthy that 60% of HCM patients with a positive family history have at least one of the eleven known sarcomere protein-encoding genes mutations. However, only 30% of HCM patients without positive family history have them (31). These genetic differences lead to significant phenotypic diversity in HCM, affecting various heart structures (32). Our finding confirms the genetic role in different HCM phenotypic subgroups.

### Limitations

Despite its remarkable findings, the present investigation has some limitations. Firstly, the retrospective design of this study limited the data to available medical records and CMR images. Prospective studies with precise predefined clinical variables and CMR protocols may provide more reliable findings. For example, although the gold standard for LVOT gradient measurement is cardiac catheterization, we could not find the catheterization data in the medical records. Doppler echocardiography at rest and with provocative maneuvers were employed to estimate the highest LVOT gradient in our cases. However, previous studies' report of a good correlation between Doppler echocardiography estimations and cardiac catheterization results, convinced us to a great extent (33). Additionally, the use of novel CMR methods, including mapping techniques and 4D flow measurements, is warranted in the assessment of patients with HCM. Secondly, our study had a limited number of patients in each subgroup. Further large-scale multicentric investigations may provide more robust results in subgroup analysis. Finally, the number of the healthy population was a limitation in our investigation. We believe that more reliable results could be obtained by increasing age- and sex-matched healthy control subjects in future studies.

## Conclusions

Cardiac MRI-derived strain measures are valuable in revealing subclinical functional alterations in HCM patients with unimpaired EF values and low fibrosis burdens. Furthermore, CMR-FT measures, distinctly LVGCS, are powerful predictors of the outcome in this patient population. It is noteworthy that the CMR-FT method can explain alterations in LV mechanics in different myocardial mass measures.

## Data Availability

The raw data supporting the conclusions of this article will be made available by the authors, without undue reservation.
